# Absent organs—Present selves: Exploring embodiment and gender identity in young Norwegian women's accounts of hysterectomy

**DOI:** 10.3402/qhw.v10.26720

**Published:** 2015-04-30

**Authors:** Kari Nyheim Solbrække, Hilde Bondevik

**Affiliations:** Institute of Health and Society, University of Oslo, Oslo, Norway

**Keywords:** Hysterectomy, gender identity, illness narratives, suffering, heteronormativity, cultural competent healthcare

## Abstract

In this paper, we explore how younger women in Norway construct their embodiment and sense of self after hysterectomy. To do this, we conducted in-depth interviews with eight ethnic Norwegian women aged between 25 and 43 who had undergone hysterectomy. In line with a broad phenomenological approach to illness, the study was designed to explore the trajectories of the women's illness with a specific focus on concrete human experience and identity claims from a subjective point of view. In analysing the stories, we encountered feelings of suffering due to the loss of the uterus as well as profound side-effects, such as menopause. However, we also found evidence of relief from being treated for heavy bleeding and serious illness. In order to accentuate the individual voices in these illness stories, we chose a case-oriented analysis in line with Radley and Chamberlain (2001) and Riessman (2008). From this, two main seemingly contradictory storylines stood out: *They have removed what made me a woman* versus *Without a uterus, I feel more like a woman*. We also identified heteronormativity as an unstated issue in both these storylines and in the research data as a whole. Acknowledging diversity in the way women experience hysterectomy is important for a better understanding of the ways in which hysterectomy may affect women as humans as well as for developing more cultural competent healthcare services for this group.

Hysterectomy is the most common major gynaecological surgery. Yet, little is known about the way this surgery impacts women's embodiment and sense of self, especially the effect it might have on younger women. Acknowledging diversity as well as hegemonic ideas about gender and the way that hysterectomy is experienced is important for a better understanding of the ways in which hysterectomy may affect women's health and well-being as well as in developing appropriate healthcare services for this group.

Hence, in this paper, we critically explore constructions of gender and embodiment after hysterectomy. More specifically, we ask: what characterises the narratives of young ethnic Norwegian women who have had their uteruses removed? What do these stories tell about the relationship between embodiment and gender in contemporary Western culture?

## Background

Although women and men share most of the same organs, some organs are gender-specific: namely, the breasts and, most significantly for women, the uterus and ovaries. Throughout history, these female organs have also been widely regarded as the very core of femininity, as being “what makes a woman a woman.” A recent incident that received a great deal of media attention in the spring of 2013 was the American A-list celebrity Angelina Jolie's decision to have both her breasts removed. The media's reaction showed the strength of the association between breasts and femininity in our culture (Gripsrud, 2006). Jolie opted for this procedure in the absence of disease because her mother had died of breast cancer at the age of 56. Jolie wrote in the *New York Times* Readers’ Column that she had the so-called BRCA1 gene mutation, which she had inherited from her mother, and which significantly increases the risk of both breast and ovarian cancers. Her contribution to the newspaper ended with: “On a personal note, I do not feel any less of a woman. I feel empowered that I made a strong choice that in no way diminishes my femininity” (Jolie, 2013).

Through its active denial of any loss of femininity, this statement may be interpreted as a confirmation of the dominant understanding of femininity as being permanently and unambiguously embedded in the specific biological body, above all, in the parts that are notably different from those of men. It is this fundamental way of linking identity and body that most probably makes the statement “I do not feel any less of a woman” seem particularly timely. Interestingly, Jolie has recently decided to have her breasts reconstructed, which, in spite of some significant representations of mastectomy as socially honourable without wearing a protease (Lorde, 1997; Jain, 2013), demonstrates the persistent importance of displaying femininity by an acceptable body standard.

In the same way that the breast have contributed—and evidently continues to contribute—to a hegemonic cultural definition of femininity, there are cultural and social reasons to believe that the uterus is so inclined, although in a presumably more mundane way than the breast. Given the growth in new forms of reproduction in the past few years, such as assisted fertilisation and surrogacy, there is no doubt that the uterus is the nexus of these transitions. In other words, although traditional definitions of parenthood and pregnancy have radically changed and diversified into new social and biological forms, the status of the uterus as the place in which the foetus develops and is nourished remains unchanged and undisputed (Kroløkke & Pant, 2012). We should note, however, that uterine transplantation from one woman to another has been successfully carried out and has recently resulted in childbirth (Brännström et al., 2014).

There are two distinct types of hysterectomy: vaginal (removal of the uterus through the vagina) and abdominal (removal of the uterus through an abdominal incision). A further distinction is made between subtotal or partial hysterectomy (removal of the uterus only, with the cervix preserved intact); total hysterectomy (removal of the entire uterus, including the fundus and cervix, but not the ovaries); hysterectomy with bilateral oophorectomy (removal of one or both ovaries as well as the uterus) and radical hysterectomy (removal of the uterus, the cervix and the upper parts of the vagina and surrounding tissues).

There are many reasons why women undergo hysterectomy. The most common indications are fibroids, heavy bleeding, uterine prolapse, endometriosis, ovarian cysts and pain (Moen, 2004). The most common reasons are either heavy bleeding or some form of cancer. However, our questions relate more specifically to the women themselves and the possible psychological and social effects of hysterectomy. According to current medical literature, the majority of Norwegian women and their partners reported no negative impact on sexual satisfaction after abdominal hysterectomy, regardless of whether the hysterectomy was subtotal or total (Lonnee-Hoffmann, Schei, & Eriksson, 2006). However, these tendencies are most likely related to the immediate relief of being cured of serious chronic pain or cancer. The findings in Sekse's (2010) study, which was based on in-depth interviews with ethnic Norwegian women aged between 39 and 66 who had undergone hysterectomy due to cancer, give a more diverse picture. Among other responses, an increased experience of bodily alienation following hysterectomy was identified as well as a fear among those who had undergone hysterectomy due to cancer or a recurring illness. However, Sekse's study did not investigate the younger women's experience of hysterectomy, nor did it examine questions regarding women's gender identity after hysterectomy.

Hence, although inextricably linked with cultural conceptions of gender as well as its necessity in societal reproduction, other aspects of the uterus, such as the embodied and biographical aspects related to hysterectomy, have been scarcely examined. The existing research on this topic seems to be consistent in suggesting that despite the fact that hysterectomy does, to a certain extent, effect women's emotions and sense of femininity and sisterhood, for example, through the lack of social sharing of menstruation (Collis, 2010; Elson, 2002), there also seems to be a rather common feeling of relief and increased quality of life related to the experience (Cabness, 2010; Collis, 2010; Elson, 2002, 2004). In particular, Elson (2002, 2004), in her American study on the relationship between hysterectomy and gender identity, has generated some important nuances about hysterectomy. While some participants in her study stated that their bodies and lives had changed considerably after the surgical procedure, the answers to the question, “Am I still a woman?” were not as simple as “yes” or “no.” Rather, they expressed a more subtle understanding of what being a woman meant and the importance of the role played by the removal of the uterus and the ovaries, in some cases, in their experience and perception of themselves as women. All the participants held the common belief that gynaecological surgery had contributed to increased reflection on gender identity (Elson, 2004, pp. 171–172).

A recent survey in Mexico mapping attitudes to hysterectomy among three different groups: gynaecologists, women who had been through hysterectomy and women who had not had hysterectomy, concluded that although there were negative characteristics (sadness, incompleteness, irritableness) involved, there were mostly positive meanings relating to hysterectomy (Marván, Catillo-Loez, & Ehrenzweig, 2012). Interestingly, the group in the Mexican study tending to attach the most negative meanings to hysterectomy was the group of women who had not undergone the procedure. Another study conducted in Mexico found that the most negative views about hysterectomy (although most common among women with less education) were women's suppositions about male perceptions: “they believe that men would see them as different”; as not “useful as women,” or no “longer women” (Marván, Trujillo, & Karam, 2009, p. 695). Another highly significant finding is that younger women are suffering from more severe depression after hysterectomy than older women (Cabness, 2010). Hence, due to the state of the art in the field of hysterectomy, more studies on younger women in Western countries and their experience of hysterectomy are recommended to be carried out (Cabness, 2010; Collis, 2010; Sekse, 2010). Additionally, we want to point out that compared with breast cancer, which has been subject to remarkable public openness in recent decades (Ehrenreich, 2009; Johansen, 2012; King, 2006), gynaecological illnesses seem to exist at the hinterland of cultural discourses on the female body and women's health. Given so-called women's liberation in Western countries, which has led to celebrating female sexuality and the specificity of women's bodies, the silence regarding gynaecological issues and their physical, psychological and social implications is not only striking but also paradoxical (Wray, Markovic, & Manderson, 2007). This is particularly so when taking into account the effects of treatment relating to sexual activity and identity and the important implications for rehabilitation (White, Faithfull, & Allan, 2013). As a dominant part of qualitative studies on hysterectomy seems to have inquired into the procedure as the symbolic meaning of losing menstruation and the organ, we believe a greater focus on young women from a very broad phenomenological approach in combination with a gender-sensitized lens is needed.

Consequently, in this article, we focus on the lived experiences of women who have undergone hysterectomy and the interplay of embodiment and identity this experience may evoke. To contextualize our aim, we find it relevant to first have a look into the medical history of hysterectomy and the female body.

## Historicising hysterectomy

A closer historical examination of how culture in general and medicine in particular have viewed and treated the uterus brings some interesting issues to light. By way of introduction, we should mention that *hysterectomy* (surgical removal of the uterus), either total (including removal of the ovaries) or subtotal/partial (removal of the uterus only), has been performed for both medical and psychological reasons since the nineteenth century. At that time, too, the main reasons for these procedures were cysts (malignant and benign), fibroids and heavy bleeding. Gynaecological surgery in connection with more diffuse psychological symptoms was also highly controversial in the nineteenth century. It was also during this period that women's medicine progressed from being part of general medicine or obstetrics to becoming a separate specialty—gynaecology. As an extension, gynaecological disease became closely associated with general female pathology. Gynaecology was also transferred to surgery, both institutionally and as a discipline. As Johannisson (1996) put it, just as the rate of cardiac disease increased when the stethoscope enabled its better diagnosis, female diseases became fashionable when gynaecological examination was liberated from the “stamp of secrecy,” and female organs could be acknowledged (p. 171). In addition to hysterectomy, *ovariotomy* (removal of the ovaries), *salpingotomy* (removal of the fallopian tubes), *clitoridectomy* (removal of parts of the clitoris) and *uterine repositioning* (alteration of the position of the uterus) were other therapeutic innovations introduced in the nineteenth century. These different procedures were introduced at slightly different times and on the basis of different indications.

While the term hysterectomy was mentioned in ancient Greek texts, several sources point to uncertainty regarding the extent to which such procedures were undertaken at that time as well as the indications upon which they were based. However, from the middle of the nineteenth century, with the introduction of modern anaesthetics, various forms of gynaecological surgery were increasingly used to treat different disorders of the female reproductive organs (Baskett, 2005). The first subtotal abdominal hysterectomy (through an abdominal incision) is said to have been performed in 1843 in Manchester by the English doctor Charles Clay to relieve the effects of fibroids in the uterus (Baskett, 2005; Sutton, 2010). The surgery was considered a success, but the woman died 5 days after the operation. The first patient to survive subtotal hysterectomy underwent the procedure in 1853. This procedure also involved hysterectomy through an abdominal incision. Vaginal hysterectomy is an older method and is the one referred to in the oldest texts. The first total hysterectomy was performed in 1929. Prior to this, subtotal abdominal hysterectomy was standard, but this was replaced by total abdominal hysterectomy during the 1950s. Over time, new techniques in anaesthesia, improved surgical methods, the availability of blood transfusions, and the discovery of antibiotics have enabled hysterectomy to become the second most common form of surgical procedure performed at women's clinics and gynaecological departments (Baskett, 2005). More recently, the development of laparoscopic hysterectomy in the 1990s has once again helped to make vaginal hysterectomy the preferred technique.

In the late-nineteenth century—and most relevant for our concern on gender and identity—it was thought that the uterus and ovaries controlled women's minds from puberty until menopause. Several doctors insisted that the instability of women's fertility organs influenced their sexual, emotional and rational control (Elson, 2004; Showalter, 1987, p. 55). According to Dally (1991), hysterectomy was thus also performed on the basis of diagnoses of hysteria, melancholy, onanism, overeating, and suicidal tendencies. These procedures were undertaken despite the 50% death rate recorded from 1881 to 1885 (Dally, 1991, p. 220). The justifications for hysterectomy changed at the beginning of the twentieth century and, similar to today, were primarily associated with abdominal pain and cancer. However, there is reason to emphasise that, at least as far as Norway (the country of focus of this study) is concerned, hysterectomy was probably not widely practised on the basis of so-called mental disorders. As discussed by Bondevik (2007) in her work on hysteria in Norway in the late-nineteenth century, the medical scientific literature showed a rather restrictive approach to hysterectomy on the basis of psychological symptoms (Bondevik, 2007; Roll, 1867). According to Lie, who studied menstrual bleeding in the same period, Battey's surgery (removal of healthy ovaries) was not performed extensively in Norway (Lie, 2012).

As shown above, neither the body and its specific organs nor the way that medicine treats disease exists in a cultural and historical vacuum. Using these insights as an important backdrop, we enquire more specifically in the following sections about hysterectomy on a personal level in present-day Norway.

## Methods

This article is based on an interview study of ethnic Norwegian women who had experienced hysterectomy over the last 3 years. In line with a broad phenomenological approach to illness and suffering (Frank, 1991, 1994; Kleinmann, 1988; Svenaeus, 2011), the study was designed to explore the trajectories of the women's illness with a specific focus on concrete human experience and identity claims from a subjective point of view. Approval was obtained from the Regional Committee for Medical and Health Research Ethics. We initially planned to recruit participants via contacts in gynaecology clinics; however, due to the ethical considerations regarding matching treatment numbers with actual persons, we decided to scrap this strategy and instead approached the Norwegian Gynaecological Cancer Association. This turned out to be a fruitful strategy as we were able to benefit from the association's help in disseminating information about the project and its aim. In total, we conducted eight in-depth interviews with women aged between 25 and 43. While some had recently undergone hysterectomy, for others, some years had lapsed between the surgery and the interview (up to a maximum of 3 years).

The interviews lasted approximately 2 h, were audiotaped and then transcribed. Six of the eight interviews were conducted by both authors and took place in a quiet area of the university where the researchers are based; one interview took place at a participant's home and one at a participant's workplace. At the start of the interview process, we brought with us an interview guide based on some key issues that our literature review and theoretical approach had brought to the fore. However, as the first of the female participants eagerly and trustfully talked through “her whole story,” and we consequently rapidly expanded our understanding of the complexity and variations of hysterectomy, the interview guide was downplayed in favour of a more open and dynamic approach. Thus, very early on, letting the story unfold from the interviewee's point of view became our main approach. Since almost none of the participants had previously told their stories in full, several of them also expressed explicit approval of our interest in their personal journey through illness.

This is not to say that the atmosphere during the interviews was easy going. As most of the women had profound, personal illness trajectories to tell, and as most were for the first time “told in total,” we as interviewers shared their human suffering. At times, this touched us deeply and made us clearly aware of our own bodily being and vulnerability (Engelsrud, 2005; Finley, 2002). We also noted the humility shown by the participants and their gratitude for having survived serious illness. As some of them made use of humour and irony in their narratives, there were also episodes when we laughed and smiled together. Our main point here is that because hysterectomies are often carried out due to serious illnesses, such as ovarian cancer which has the highest death-to-case ratio among all malignancies, a dialogue about these issues also entails dealing with the scenario of early death (Bowes, Tamlyn, & Butler, 2002).

To investigate the relationship between the body and gender identity in the actual stories, which were co-produced by the interviewees, we chose to analyse them using a narrative approach to illness and health. In the literature, narratives are often claimed to demonstrate what is uniquely individual (Charon, 2006), which is also always related to the body, and to provide greater insight into cultural codes and historical patterns that are typical of their time (Frank, 2013; Riessman, 2008). As a result, we found the narrative approach most useful for our analysis. Furthermore, in understanding illness narratives as stories displaying how the outer world appears from the teller's viewpoint, the analysis identified a range of new dimensions in these women's lives following hysterectomy. Taking a step further and relating these dimensions to aspects of the body and gender identity, two main seemingly contradictory storylines regarding these dimensions stood out: *They have removed what made me a woman* versus *Without a uterus, I feel more like a woman*. In order to accentuate the individual voice in these illness stories, we chose a case-oriented analysis, in line with Radley and Chamberlain (2001) and Riessman (2008). Put differently, we used what we heard from the informants during the entire research process to drive our understanding of what was the most significant research question, and from that, we selected some cases of individual experience that illuminated the question in a lively and vivid way. In sum, there was an active use of *phronesis* in the process of analysis (Frank, 2010). After presenting the main sequences constituting the two main storylines, we more broadly discuss their implications. At this point, we discuss heteronormativity as a taken-for-granted social category on which these women's post-hysterectomy constructs of gender and identity seem to be embedded.

## Findings

### “They have removed what made me a woman”

This story is based on those parts of the narratives in which the link between the loss of the uterus, on the one hand, and the loss of the personal experience of being a full-fledged woman, on the other, are expressed in an incontestable and causal manner. Sandra's narrative about undergoing hysterectomy demonstrates this link in a vivid and specific way. We have therefore chosen to use her story to represent the first main storyline in this paper. Sandra is 24 years old, and the following excerpt is her account of how her and her partner's plans to have children were abruptly reversed. The broader background of this experiences as such a radical and abrupt reversal was that Sandra was being assessed for surgery over a fairly long period due to radical cell mutations and suspected cancer, which her doctors had believed was under control for a long time. Then, the blow came:

We'd been told that next time, if all the tests were fine, we'd get the thumbs up to try for children again. And I was full of expectation. I'd also lost 15 kilos and had regular periods, so everything looked really good. Then I got a letter in the post in May, in the middle of May, that I had had a relapse. And that was tough. Yes, tough. I'd always dreamed of the day I could be there with a positive pregnancy test in my hand, an ultrasound, a heartbeat, yes, all that sort of thing. So it was very tough. The evening I got the letter, I collapsed and screamed, and the neighbour came to ask what had happened.

So it was a shock; it really was. But in a way, I'd somewhat prepared myself for it as well. Because they always said, “It might happen that you can't have children, that we'll have to remove your uterus, but for now, we'll see how the treatment goes and whether you react well to it.” So yes, it was a shock. I do have a lot of friends who are now pregnant or trying to get pregnant, and there's a lot of talk about children and things like “I want this number of children, and they'll be called this and that.” So it's hard; it really is. But I did keep my ovaries, at least, so I avoided going into menopause, and there's the possibility of surrogacy.

For us, this sequence from Sandra's story depicts with immense clarity the biographical disruption (Bury, 1982) the feeling of homelessness (Svenaeus, 2011) and self-othering (Halliday, Broughton, & Kerridge, 2014) that hysterectomy, in the context of serious illness and lack of reproductive capabilities, may represent. Staying with Sandra's narrative a little longer, the wider effect of the procedure on her partner and on their relationship is clearly significant:

He's said that children are not really the most important thing for him. The most important thing for him is that I'm healthy, and that's very true. But when I got the news that I had to have a hysterectomy, I said to him, “I know how important it is for me to be able to have a child, a biological child, so if you want, you can leave. I'll never be angry about it or hold it against you because I know how important it is for me.” And I remember that I said that several times. But he said: “I love you, not because you can give me children, I love you because of who you are.” But I still feel that in a way, I robbed him of the opportunity to be able to experience that. And now we have problems in our sex life, and it is he who doesn't want to. I want to, but he doesn't. So we're trying to make sense of everything that's happened. And I think that it [the problem] may be that he's afraid or has lost interest because I can't have children. Yes, because it can't result in a child. We're going to see a psychologist now to talk about it. So it's got a bit more complicated. It really has. And I feel guilty about it. Yes, I do.

As the excerpt demonstrates, Sandra's experience of hysterectomy includes not only her own self and her ideas of the future. The way in which she articulates her partner's reactions and the feelings of guilt associated with his sexual withdrawal also bear witness to the profound existential and relational effect that may be caused by hysterectomy of this kind, a point substantiated in a recent study (Askew & Zam, 2013). In more general terms contours of the moral boundaries in which the “cared for” and the “carer” ontologically are embedded in, comes to the fore (Chattoo & Ahmad, 2008).

In the final sequence from Sandra's story, a fundamental doubt about the meaning of life and, moreover, her gendered identity is also brought to the fore:

In the first period after the surgery, I thought, okay, I chose life, but I also rejected the meaning of life. I've worked on it quite a bit, on the grief, in the past year. Nevertheless, sometimes I can feel it now too: What is the meaning of my life now? The meaning of life for me was becoming a mother, experiencing childbirth, experiencing the feelings that a couple shares through pregnancy and birth.

In addition, I've felt less feminine. They have, in a way, removed what made me a woman, what distinguishes a man from a woman. But then I thought, “Thank God, I have a partner! Because if I didn't have a partner, I would certainly have ended up alone. I can't give them [men] a child. And that definitely makes me less of a woman, for them, yes.

Sandra's account of hysterectomy appears to resemble a tightly woven fabric of three elements: an absent organ, human suffering and a fractured gender identity, or to be more specific, the notion is that the uterus *is* the very incarnation of femaleness and of the person she wants herself to be. The fact that recovery from serious cancer, as in Sandra's story, seems to be downplayed against the human suffering of not having one's uterus intact demonstrates how deeply disrupted some women's sense of self and life-expectancy may become after hysterectomy.

### “Without a uterus, I feel more like a woman”

In our study, however, there were other accounts in which these elements, taken together, point to a different outcome. Karen's narrative, in particular, demonstrates the opposite of that recounted by Sandra and is therefore chosen as our second main case. The specific background to Karen's hysterectomy was heavy bleeding. However, it took 8 years from the time she first asked her doctor to consider her for a hysterectomy until she got the green light. In contrast to Sandra, therefore, Karen fought hard to undergo hysterectomy and to be believed that she genuinely thought it was the best thing for her well-being. Karen is in her late thirties, and unlike Sandra, she has biological children. Problems with her uterus began in earnest after the birth of her last child:

It started when I had my last child. At that time, I also had a lot of problems with bleeding, heavy bleeding. The gynaecologist said they could try to remove the endometrium, and then they could remove the uterus, but she thought I was much too young to do it then. I really wanted it done quite quickly, but it was not allowed. You see, she thought I was still too young in case I wanted more children.

I did actually have very light bleeding before that. But gradually, the bleeding became really extreme, and that's when it really began in earnest. I might get up from my seat on the train and whoosh, I just had to turn round and go back home. And it went on and on. I used to have a change of clothes with me and went around with a bag of clothes all the time. It was a bit hopeless. I was aged 36 or 37, and as I say, I would actually have liked to have everything removed. I was examined to determine whether I had a large uterus, fibroids and so on; and I did, I had an enlarged uterus. I also had some fibroids. But they didn't think this was enough to justify removing it and felt that I might regret it.

In this part of the story, we are struck by how actively the healthcare system, in spite of the suffering displayed, enforces women to preserve their uterus. The way in which Karen portrays the process displays a rich sense of paternalism towards female patients, for which the medical establishment, as previously outlined, is well known. As Karen's story unravels, the relational consequences of this refusal by the medical establishment to help her become clear:

I've been single for a long time, you see, and it is a very poor basis for meeting someone when you are running around having your period the whole time and are not able to be sexually active. You're leaking blood, you can't cope with it; you hesitate to socialise too because you never know how it will be. So you then become a bit unsociable, actually. That's a little strange because I am actually a very sociable person. So maybe you make the excuse that you're alone, and you stay at home and you don't participate in things. So it kind of limits your social life; you are always wearing black trousers, even in summer; you have dark brown bed linen; you don't have those airy, light, bright colours. So it limits you in a way.

So far, Karen's story not only displays bodily suffering but also clearly demonstrates the intense personal and social issues that having constant uterine bleeding represents. However, after years of struggling to have a hysterectomy, her needs were eventually acknowledged:

In the end, I demanded it myself. I went to my doctor and said I wanted a hysterectomy because then I was over 40, and then, according to another gynaecologist I spoke to, you can be more certain of the choice you have made not to have more children. It was really a relief to have done it. I'd do it again, and I recommend [to] anyone who has had enough children to do it. Since the bleeding stopped, I got a whole new life. My ferritin levels rose, but then my weight started to increase too, and I wasn't informed that that could happen, so that was a disadvantage. But I would have done it; I would have had the hysterectomy anyway because aside from the weight gain, I got rid of all the symptoms. Other people notice it too that I have more energy for things. Now I can go to the indoor swimming pool; I can start to exercise and go for walks because I didn't do any of that back then.

I actually feel a lot more feminine now, a bit more of a free woman, a bit more liberated. I'm not so tied to those cycles as I was before. So as far as I'm concerned, I'm a lot freer now. And once you've made the choice that you don't want any more children, the fertility itself, you no longer need it. It was just a burden.

I feel much freer and more feminine now since I can now wear what I want, and I don't have to run around with an extra bag of clothes to drag along; I just don't have to plan so much around myself. I always had a change of clothes with me, always a bag of clothes in the car. During the worst times, I slept wearing baby nappies, the biggest and heaviest you can get.

It was really and truly awful. I'd be at work for seven or eight minutes, and then I'd have to stop and change my towel.

We then asked, “Do you think that in the future it will be complicated to tell a partner that you have had a hysterectomy?”

No. Not when I'm the age I am now. Because, how shall I put it: a potential partner for me may possibly have children, or he won't be interested in having children. If I'd been younger, it might have been a problem if I'd had it removed too early.

As this narrative unmistakably shows, undergoing hysterectomy may lead to a definite improvement to a woman's quality of life. As for the question of gender, getting rid of the bleeding and its social implications also open the way to defining oneself as a far more liberated woman than before. Admittedly, Karen already has children, but it is nonetheless striking how she, in contrast to Sandra, separates femininity from the uterus and the inherent capacity to bear children. In doing so, she also explicitly turns upside down the traditional cultural bonds between specific bodily organs and being female.

## Discussion

As mentioned earlier, the uterus has historically been central to any understanding of what a woman is and should be. Based on our study of young Norwegian women's hysterectomy narratives, there is no doubt that the effects are significant in relation to embodiment and sense of self. In analysing the stories, our interest was aroused by two particular storylines. In one storyline, hysterectomy clearly represents liberation from continuous heavy uterine bleeding with its attendant serious social consequences. For other participants, this procedure means that they are cured of a serious illness but must in turn suffer the loss of not being able to bear children, thus being left in an echoed silence (Johansson, Axelsson, Berndtsson, & Brink, 2014). These variations resonate with other findings (e.g., Cabness, 2010; Collis, 2010; Elson, 2002), and in particular, they point to the fundamental consideration that a woman's stage in life clearly influences the way she experiences hysterectomy.

Reflecting more fundamentally on these findings, the question of what constitutes femininity in our Western culture comes to the fore. Is it a biologically-based capacity to experience pregnancy and consequently motherhood as Sandra's narrative so strongly indicates? Conversely, is it about having a clean and socially presentable body as Karen's story illustrates? Linking these questions to a discussion on how to best designate healthcare services for women undergoing hysterectomy gives rise to additional reflections. Should society take account of these aspects of body and gender when organising health and rehabilitation services for this group of women, and if so, how should these services be designed and implemented (Wijma, Smirtswaite, & Swahnberg, 2010)? From another vantage point, are women's understandings of their body and identity a cultural stereotype or a copy of an imagined nature (Butler, 1990) that ought to be neglected or even argued against in the context of illness and care?

As the analysis above has demonstrated, constructions of gender identity after hysterectomy are diverse, thus calling for serious reflection on the topic. In our analysis, we have highlighted two quite different, if not opposite, positions articulated by the afflicted women themselves. Given that the uterus for some might appear as “the object” for seeing oneself as a worthy individual, losing it could act as a rupture in women's lives and self-perception, and this sense of damage should not be neglected or silenced by their clinicians or next of kin. This situation seems particularly true for women who have not had children and who have envisaged this as a key part of their future lives: “Who am I now?” Nevertheless, viewed in the context of history, in which the fight for greater social and political rights for women has been pivotal, the persistent status of the uterus and biological motherhood in relation to defining women's self-image is thought-provoking, if not paradoxical. Even with the expansion of free abortion services and lifestyle choices other than the traditional heterosexual nuclear family—be it the single life, same-sex partnership or the intimacy of friendship—the choice of biological motherhood and its importance for women's identity does not appear to be seriously contested in Norway (Ravn, 2005). Regarded as a discursive phenomenon rather than a biological entity (Foucault, 1990; Laqueur, 1990), the seemingly signifying effect the uterus appears to have on gender identity—such as in Sandra's case, is also striking. Perhaps the notion of an inner room creating one's self as well as one's outer space, is more powerful than what the modern discourses on gender often put on the agenda.

The discussion also easily leads us to an overall feminist theoretical landscape in terms of how to conceptualize the intersection of gender and embodiment. On a general level, it is tempting to state that with the post-structural turn in feminist thinking, led by Butler's (1990) heavy stress on gender as something one *does* rather than something one *is*, research on the possible intersections of the specific female body (morphology) and social identity has been remarkably halt (Lykke, 2008). However, more subtle perspectives on body and gender have been developed, such as the post-material body (Ahmed, 2008; Barad, 2007), and in the context of social science and medicine, several feminist scholars have argued for a position which we find most relevant in the field of gender and health, that is, “a synthesis of both biomedical and social constructivist perspectives in order to capture the complex, subjective and embodied nature of female response in both health and illness” (White, Faithfull, & Allan, 2013, p. 188).

Hence, part of what we believe that our study on hysterectomy captures is that even though we are concerned about women's health and embodiment, we should very clearly avoid biological essentialism. As Karen's narrative crystallises, the possibility of living a meaningful life without a uterus—sometimes an even better one - is definitely within range. What Karen's case also illuminates is the way in which extremely heavy uterine bleeding may create a number of restrictions to a woman's working and sexual life. Having said that, we believe that Karen's struggles with heavy bleeding may also highlight a contemporary norm of being as pure as possible. One might easily believe that this norm of bodily purity has always been the case for women, but in fact, until the eighteenth century, women's blood was considered necessary to balance their health (Finucci & Brownlee, 2001; Lie, 2012). In analysing the stories, we encountered feelings of suffering due to the loss of the uterus as well as other side-effects of hysterectomy, such as menopause, which is an aspect of corporality. In this sense, we can argue that female bodies, menstruating or not, are confronted today with refined requirements for purity and perfection. In fact, one of the most striking features of youth culture is the desire to control or minimise menstrual bleeding (Oinas, 2001). At the same time, at least in Norway, society is simultaneously characterised by a widespread expectation of biological motherhood (Ravn, 2005). Consequently, the ideal of becoming a mother while simultaneously minimising fluids such as blood represents a powerful cultural paradox. Perhaps it is in this cultural tension between a powerful modern norm of displaying a pure and publicly presentable body, on one hand, and the deeply internalised desire for a fertile body, and therefore also a bleeding one, on the other, that we can best understand what women's stories of hysterectomy are all about.

Focusing on the clinicians’ advice to Karen over the years also implicates the medical establishment and its persistent preoccupation with the uterus as an absolute necessity that represents the value of being a woman. By this form of praxis, alternative definitions and queer experiences of illness and the female body (Jain, 2013), for instance, appear to be completely neglected or silenced—a social mechanism which coincides with White's study on how women's sexuality after pelvic radiation is mainly reconstructed within an essentialist and heteronormative oncology (White, Faithfull, & Allan, 2013). A closer look at the narratives in our study reveals this to be an important underlying logic in all the stories; Sandra's and Karen's accounts included and are told as the outer world without doubt *is* heterosexual. As a result, when the personal experience of hysterectomy, the medical reasons for it as well as the treatment trajectories are exclusively framed in this way, the illness trajectories experienced by women who have female partners or who are not heterosexual (Hyde, 2007; Jain, 2013) are effectively silenced. An acknowledgement and further inquiry into this aspect are significant for a better understanding of the sense of self and embodiment that hysterectomy may lead to, and on these grounds, the development of a more nuanced and culturally-appropriate healthcare service.
